# Mean platelet volume to platelet count ratio as a laboratory indicator of mortality in pneumonia following ischemic stroke

**DOI:** 10.1186/s41983-018-0028-9

**Published:** 2018-09-26

**Authors:** Rania S. Nageeb, Mohammed M. N. Abozaid, Ghada S. Nageeb, Alaa A. Omran

**Affiliations:** 1 Department of Neurology, Faculty of Medicine, Zagazig University, Sharkia, Egypt; 2 Department of Chest, Faculty of Medicine, Zagazig University, Sharkia, Egypt; 3 Department of Rheumatology & Rehabilitation, Faculty of Medicine, Zagazig University, Sharkia, Egypt; 4 Department of Clinical Pathology, Faculty of Medicine, Zagazig University, Sharkia, Egypt

**Keywords:** Mean platelet volume to platelet count (MPV/PC) ratio, Acute ischemic stroke, Pneumonia and mortality

## Abstract

**Background:**

Platelets have a vital role in antimicrobial host defenses. The objective of this study was to evaluate if increased mean platelet volume to platelet count (MPV/PC) ratio in acute ischemic stroke patients complicated with pneumonia was associated with increased mortality risk.

**Methods:**

The current study was conducted at Zagazig University Hospitals. It included 500 acute ischemic stroke patients classified as group 1 that included 51 patients complicated with pneumonia after admission and group 2 that included the remaining 449 patients. Clinical assessment was carried out to exclude comorbid medical illnesses likely to interfere with platelet function or morphology. Laboratory investigations including MPV/PC ratio and brain imaging were carried out for all patients.

**Results:**

There was a significant difference between both groups regarding age, National Institutes of Health Stroke Scale (NIHSS) score, and mortality within 30 days (*p* = 0.02, 0.03, 0.01). There was a significant difference between survivors and non-survivors of group 1 regarding to pneumonia severity index (PSI) classes IV and V (*p* = 0.01 and 0.02, respectively). Also, there was a significant difference regarding confusion, urea ≥ 7 mmol/L, respiratory rater ≥ 30 breaths/min, systolic blood pressure ≤ 90 mmHg or diastolic blood pressure ≤ 60 mmHg, and age ≥ 65 years at pneumonia occurrence (CURB-65) scores 3, 4, and 5 (*p* = 0.03, 0.02, and 0.01, respectively). Moreover, there was a significant difference regarding decreased GCS score at pneumonia occurrence, higher NIHSS scores, PSI, and higher MPV/PC ratio (*p* = 0.01, 0.01, 0.028, and 0.01, respectively). Age > 65 years, need for mechanical ventilation, GCS score of > 9, PSI class ≥ IV, CURB-65 scores ≥ 3, and increased MPV/PC ratio were all significantly associated with 30-day mortality in group 1 (*p* = 0.03, 0.01, 0.001, 0.04, 0.01, and 0.03, respectively). The predictors of 30-day mortality risk factors were GCS less than 9, increased MPV/PC ratio, and CURB-65 scores ≥ 3 (*p* = 0.001, 0.05, and 0.01, respectively).

**Conclusions:**

Once pneumonia develops, MPV/PC ratio could be considered a significant laboratory indicator of 30-day mortality.

## Background

Stroke is a major cause of disability and death worldwide (Pinto et al. 2014). About one third of stroke patients suffer from pneumonia, and this increases the morbidity and mortality of these patients (Sellars et al. 2007). Pneumonia causes the highest attributable mortality of all medical complications following stroke (Alberti et al. 2011).

Stroke causes neurogenic dysphagia that besides limiting food intake, it carries a greater risk of malnutrition and tracheal aspiration and triples the incidence of aspiration pneumonia (Baroni et al. 2012). This higher risk is due to the possibility of silent aspiration (without cough reflex), micro aspiration, impairment of the laryngeal closure mechanism, delayed swallow initiation, motor dysfunction of the pharynx, presence of pharyngeal residues at epiglottic valleculae, pyriform sinuses, and posterior wall of the pharynx (Nunes et al. 2012).

Platelets have been recognized as an essential component of innate and adaptive immunity (Fitzgerald et al. 2006). Platelets contain antimicrobial peptides that exert a rapid, potent, and direct antimicrobial effect that contributes to limiting the infection (Yeaman and Bayer 2006). Thrombocytopenia is a recognized marker of poor outcomes in hospitalized patients with pneumonia (Mandell et al. 2007).

Higher values of mean platelet volume to platelet count (MPV/PC) ratio have been established in patients with different subtypes of stroke than in controls. Furthermore, mean platelet volume (MPV) has been shown to be predictive of stroke, in patients with previous cerebrovascular events. These findings lead to the hypothesis that the increase of MPV/PC might have a critical role in the outcome of stroke (Elsayed and Mohamed 2017).

The aim of the work is to investigate the association of increased mean platelet volume to platelet count (MPV/PC) ratio with increased mortality in hospitalized acute ischemic stroke patients complicated with pneumonia.

## Methods

This is a cross-sectional study that was done in the period from April 2015 to March 2017. All patients meeting the inclusion criteria of this study and admitted in the stroke units of Neurology, and Internal Medicine departments in Zagazig University Hospitals, Sharkia Governorate, Egypt, during this period were included in the study. Five patients died within 3 days after admission out of 505 patients, so only 500 were included in the study, and the patient’s age (mean ± SD/years) was 67.3 ± 10.2. Fifty-one out of 500 complicated with pneumonia, those patients were classified as group 1 (37 of them were males and 14 were females), and 449 were not complicated with pneumonia, those patients were classified as group 2 (68% of them were males and 32% were females).

### Ethical consideration

A written consent was taken from all of the participants or their relatives after explaining the details of the study to them. The study was approved by the Institutional Ethical Committee of Faculty of Medicine, Zagazig University, Egypt (ZU-IRB#4728\24-3-2017).

#### Inclusion criteria

Patients were eligible for inclusion in the study if they had evidence suggesting the following:

1. Acute ischemic stroke. Stroke was diagnosed clinically (as there was a new onset of neurological deficits that is corresponding to a vascular origin in the brain and lasted for more than 24 h) and proved via brain imaging (computed tomography with or without magnetic resonance imaging) (Chen et al. 2013).
2. Pneumonia following acute ischemic stroke during the same hospitalization. Pneumonia was diagnosed if the acute ischemic stroke patient had relevant clinical manifestations (fever, new or increased cough, purulent tracheal secretion, or leukocytosis), positive microbiologic findings (blood, sputum cultures), and new onset of pulmonary infiltrates on chest radiography (Mirsaeidi et al. 2010).

#### Exclusion criteria

Patients were excluded from the study if they had comorbid medical illnesses likely to interfere with platelet function or morphology, e.g., chronic kidney disease, chronic liver diseases, and leukemia.

All patients were subjected to the following:

1. Full history taking
2. Thorough general and neurological examination
3. Laboratory investigation

Complete blood count (CBC), using automated cell counter “model XS 500i (Sysmex, Japan),” together with examination of Leishman stained peripheral blood smears for differential leukocytic count. Mean platelet volume to platelet count ratio (MPV/PC) was calculated. MPV/PC ratio was considered increased if it is more than 0.031 according to Elsayed and Mohamed, 2016 *(*Elsayed and Mohamed 2017). Significant leukopenia and leukocytosis were defined as white blood cell (WBC) counts of, ≤ 4000 and ≥ 25,000, respectively. Thrombocytopenia and thrombocytosis were defined as platelet counts, ≤ 100,000/L or ≥ 400,000/L, respectively.

-Liver and kidney functions using automated analyzer “Cobas 501” (Roche diagnostics, Switzerland).

-C-reactive protein (CRP) and arterial blood gas analysis.

Alteration of gas exchange was defined as PaO_2_ < 60 mmHg or O_2_ saturation < 90% (Mirsaeidi et al. 2010; Katz et al. 2011).

4- Imaging to confirm the presence of acute ischemic stroke and pneumonia: brain imaging (brain computed tomography with or without magnetic resonance imaging) and chest plain X-ray.
5- Scales for assessment of patients included in the study:

- Neurological scales:

A- Neurologic status was assessed by the Glasgow Coma Scale (GCS) on admission and on pneumonia occurrence **(**Teasdale and Jennett 1974**)**. It can be elicited via assessment of eye opening, motor response, and verbal response. Total score ranged from 3 to 15.
B- The clinical severity of stroke was assessed on the day of admission using the National Institutes of Health stroke scale (NIHSS) (Lyden et al. 2001). The NIHSS is a well-validated and commonly used stroke impairment scale that is used to evaluate the level of consciousness, language, speech, extraocular movements, visual field loss, motor strength, sensory function, coordination, and hemi-neglect. We rated the patient’s ability to answer questions and perform activities. Ratings for each item are scored with 3 to 5 grades with 0 as normal. Patients were classified according to the NIHSS score into three groups: mild stroke, when the NIHSS score was ≤ 8; moderate stroke when the NIHSS score was from 9 to 15; and severe stroke when the NIHSS score was ≥ 16.

- Mortality risk was assessed by death in 30 days after hospital admission (Mirsaeidi et al. 2010).
- Scales for assessment of pneumonia:
  1. The pneumonia severity index (PSI) score is based on age; coexisting disease, abnormal physical finding, and abnormal laboratory finding upon presentation patients were assigned to the appropriate PSI risk classes (I–V). Outpatient therapy should be considered, especially for patients in classes I and II, while patient in classes IV and V should be hospitalized (Fine et al. 1997).
  2. The CURB-65 score is based on the presence of confusion, urea ≥ 7 mmol/L, respiratory rater ≥ 30 breaths/min, systolic blood pressure ≤ 90 mmHg, or diastolic blood pressure ≤ 60 mmHg, and age ≥ 65 years at pneumonia occurrence. Patients were assigned to the appropriate CRB-65 score (1–5). A single point is given for the presence of confusion, a blood urea nitrogen value ≥ 20, a respiratory rate ≥ 30, a systolic blood pressure < 90 mmHg, or a diastolic blood pressure ≤ 60, and an age ≥ 65 years. If the score is 0 or 1, then most likely this patient could be safely treated as an outpatient. A score of 2 might suggest closely supervised outpatient treatment or inpatient observation admission. A score of 3, 4, or 5 would usually indicate inpatient treatment (Aliberti et al. 2009).

### Statistical analysis

Descriptive statistical methods were used to calculate means and standard deviation (SD). For comparisons with the continuous variables, Student’s *t* test and ANOVA were used. Comparison of categorical data was performed using the χ^2^ test and the Fisher exact test. Multivariate and multiple regression analyses were used. Statistical significance was made at *p* value < 0.05. Data were analyzed using statistical package of social science, version 14 software package (SPSS Inc., Chicago, IL, USA) (Levesque 2007).

## Results

The patients were classified into two groups; their age (mean ± SD/years) was 67.3 ± 10.2 and 59.2 ± 25.3 years, respectively. Twenty-four (53%) patients died out of group 1, and 67 (15%) patients out of group 2 died within 30 days of admission.

There was a significant difference between group 1 and group 2 regarding age (*p* = 0.02), National Institutes of Health Stroke Scale score, and number of cases that died within 30 days of admission (*p* = 0.03 and 0.01, respectively) (Table 1).

**Table 1:** Comparison between group 1 and group 2 regarding the demographic data, comorbidity and neurological scales as well as mortality risk

Risk factors	Group 1 (patients with pneumonia)	Group 2 (patients without pneumonia)	*p* value
	51 (10%)	449 (90%)	
Age (year)	67.3 ± 10.2	59.2 ± 25.3	0.02^*^
Sex, *N* (%)			
Males	37 (73)	305 (68)	0.65
Females	14 (27)	144 (32)	
Smoking	20 (39)	166 (37)	0.41
Comorbidity			
Hypertension	36 (71)	323 (72)	0.32
Diabetes mellitus	15 (29)	139 (31)	0.73
COPD	6 (12)	72 (16)	0. 51
Neurological scales			
GCS on admission	11.0 ± 3.0	12 ± 3.0	0.14
NIHSS	17.7 ± 5.9	10.2 ± 5.1	0.03^*^
Mortality risk			
Death within 30 days of admission	24 (53)	67 (15)	0.01^*^

*COPD* chronic obstructive pulmonary disease, *GCS* Glasgow Coma Scale, *NIHSS* National Institutes of Health Stroke Scale

*Significant

Group 1 included 51 (10.2%) patients (those who were complicated with pneumonia). Twenty-four (53%) patients out of them died within 30 days of admission. There was a significant difference between survivor patients and non-survivors of group 1 regarding PSI classes IV and V (*p* values were 0.01 and 0.02, respectively). Also, there was a significant difference regarding to CURB-65 classes III, IV, and V (*p* = 0.03, 0.02, and 0.01, respectively) (Table 2).

**Table 2:** Number and percentage of survivors versus non-survivors of group 1 regarding PSI and CURB-65 scales

Scale of assessment	Survivors patients with pneumonia 27 (53%)	Non-survivors patients with pneumonia 24 (47%)	*p* value
PSI			
Class І	8 (30)	0 (0)	0.001
Class Π	7 (26)	5 (22)	0.12
Class III	6 (22)	4 (20)	0.14
Class IV	3 (12)	6 (23)	0.01*
Class V	3 (12)	9 (36)	0.02*
CURB-65			
Score 1	5 (18)	0 (0)	0.14
Score 2	8 (30)	6 (25)	0.13
Score 3	8 (30)	9 (37)	0.03*
Score 4	5 (18)	6 (25)	0.02*
Score 5	1 (4)	3 (13)	0.01*

*PSI* pneumonia severity index

*Significant

Multivariate analysis comparing survivors versus non-survivors of acute ischemic stroke patients who developed pneumonia (group 1), according to risk factors of 30 days mortality, there was significant difference regarding lower GCS score at pneumonia occurrence, higher National Institutes of Health Stroke Scale scores, PSI, and higher MPV/PC ratio (*p* = 0.01, 0.01, 0.028, and 0.01, respectively) (Table 3).

**Table 3:** Multivariate analysis comparing survivors versus non-survivors of group 1 according to risk factors of 30 day mortality

Risk factors of 30-day mortality	Survivors of group 1 (*n* = 27)	Non-survivors of group 1 (*n* = 24)	*p* value
Time from stroke to pneumonia onset/days	9 ± 2	12 ± 4	0.921
Scales for assessment of ischemic stroke			
GCS on admission	12 ± 3	11 ± 3	0.461
GCS at pneumonia occurrence	11 ± 3	8 ± 2	0.01*
NIHSS	9 ± 4	18 ± 6	0.01*
PSI scales for assessment of pneumonia severity	92 ± 36	124 ± 37	0.028*
Clinical findings			
Body temperature (°C)	37 ± 1	38 ± 0.8	0.580
Respiratory rate	23 ± 5	26 ± 6	0.059
Heart rate	70 ± 20	75 ± 15	0.376
Systolic blood pressure	149 ± 20	130 ± 30	0.146
Laboratory results			
WBC count/mm3	11,641 ± 4199	10,885 ± 3960	0.583
MPV/PC ratio	0.030 ± 0.607	0.06 ± 0.01	0.01*
Blood urea nitrogen, mg/dL	25 ± 14	30.5 ± 21	0.382
Serum creatinine, mg/dL	1.5 ± 1	2.0 ± 3	0.466

*GCS* Glasgow Coma Scale, *NIHSS* National Institutes of Health Stroke Scale, *PSI* pneumonia severity index, *MPV/pc ratio* mean platelet volume/platelet count ratio

*Significant

In multiple regression analysis for 30-day mortality risk in group 1 age > 65 years, need for mechanical ventilation, GCS score of less than 9, high-PSI risk class ≥ IV, CURB-65 scores ≥ 3, and increased mean platelet volume/platelet count ratio were all significantly associated with 30-day mortality (*p* = 0.03, 0.01, 0.001, 0.04, 0.01, and 0.03, respectively) (Table 4).

**Table 4:** Multiple regression analysis for predictors of 30-day mortality in group 1

Predictors of 30-day mortality	Group 1		95% confidence intervals	*p* value
Age > 65 years	Non-survivors	12 (54%)	0.833–0.987	0.03*
	Survivors	12 (46%)		
Sex (males/females)	Non-survivors	19 (51%)/18(49%)	0.63–1.34	0.34
	Survivors	8 (52%)/6(48%)		
Smoking	Non-survivors	12 (60%)	0. 13–1.25	0.25
	Survivors	8 (40%)		
Mechanical ventilation	Non-survivors	8 (33%)	0.35–0.26	0.01*
	Survivors	6 (22%)		
GCS at pneumonia occurrence < 9	Non-survivors	15 (63%)	11.12–21.30	0.001*
	Survivors	10 (37%)		
Pleural effusion in CXR	Non-survivors	11 (46%)	0.47–1.38	0.99
	Survivors	12 (44%)		
Pulmonary infiltrates in CXR	Non-survivors	11 (46%)	0.36–1.14	0.224
	Survivors	12 (44%)		
PSI risk class ≥ 4	Non-survivors	15 (63%)	1.095–2.364	0.04*
	Survivors	6 (22%)		
CURB-65 scores ≥ 3	Non-survivors	18 (75%)	1.23–2.99	0.01*
	Survivors	14 (52%)		
Increased MPV/PC ratio	Non-survivors	16 (67%)	2.9–2.23	0.03*
	Survivors	8 (30%)		

*GCS* Glasgow Coma Scale, *NIHSS* National Institutes of Health Stroke Scale, *CXR* chest radiograph, *PSI* pneumonia severity index, *MPV/pc ratio* mean platelet volume/platelet count ratio

*Significant

In logistic regression analysis for detection of 30-day mortality risk in non-survivors of group 1, the most significant 30-day mortality risk factors were GCS < 9 at pneumonia occurrence, increased MPV/PC ratio, and CURB-65scores ≥ 3 (*p* = 0.001, 0.05, and 0.04, respectively) (Table 5).

**Table 5:** Logistic regression analysis for detection of 30-day mortality risk in pneumonia complicating ischemic stroke patients in non-survivors

Risk factor of 30-day mortality	95% confidence intervals	*p* value
GCS < 9 at pneumonia occurrence	1.25–2.35	0.001*
Increased MPV/PC ratio	2.13–2.138	0.05*
CURB-65 scores ≥ 3	1.43–1.93	0.01*

*GCS* Glasgow Coma Scale, *MPV/pc ratio* mean platelet volume/platelet count ratio

*Significant

## Discussion

Although mortality is encountered in pneumonia following ischemic stroke, there is no specific laboratory investigative tool for this entity (Chen et al. 2013). So the aim of the present study was to investigate the association of increased mean platelet volume to platelet count (MPV/PC) ratio with increased 30-day mortality in hospitalized acute ischemic stroke patients complicated with pneumonia.

There was a significant difference between group 1 and group 2 regarding age, National Institutes of Health Stroke Scale score (NIHSS), and number of cases that died within 30 days of admission. This is in agreement with a previous study of Mirsaeidi and colleagues (Mirsaeidi et al. 2010) who found that age > 65 years was significantly associated with 30-day mortality risk in patients with pneumonia.

Thirty day mortality was significantly associated with PSI risk classes ≥ IV and CURB-65 scores ≥ 3 in our ischemic stroke patients with pneumonia, and these results are consistent with Mirsaeidi and colleagues (Mirsaeidi et al. 2010) and Chen and colleagues (Chen et al. 2013). Also, Golcuk and colleagues (Golcuk et al. 2015) confirmed that the CURB-65 score could independently predict mortality in patients with acquired pneumonia.

Moreover, in the present study, patients who survived from pneumonia had statistically significant better scores in GCS score at pneumonia occurrence, NIHSS scores, PSI score, and MPV/PC ratio than non-survivors. This is in agreement with the findings of Chen and colleagues (Chen et al. 2013) who stated that lower GCS scores on the day of pneumonia onset and higher NIHSS scores were significant risk factors for 30-day mortality in patients complicated with pneumonia following acute ischemic stroke. Also, these findings met with the findings of Elsayed and Mohamed (Elsayed and Mohamed 2017) who found that ischemic stroke patients had significantly higher MPV/PC ratio that could be used as surrogate laboratory markers for early detection and risk stratification of cerebrovascular stroke. Bharosay and colleagues (Bharosay et al. 2016) stated that steady-state inverse, and nonlinear relationship between MPV and platelet count (PC), has been observed.

The need for mechanical ventilation and the presence of thrombocytosis were significantly associated with 30-day mortality in patients with acquired pneumonia according to the findings of Mirsaeidi and colleagues (Mirsaeidi et al. 2010). This was concomitant with our results as the need for mechanical ventilation and the increased mean platelet volume/platelet count ratio were significantly associated with 30-day mortality in our patients.

Authors of the present study found that the most significant 30-day mortality risk factors were GCS < 9 at pneumonia occurrence, increased MPV/PC ratio, and CURB-65 scores ≥ 3. These observations were in agreement with Elsayed and Mohamed (Elsayed and Mohamed 2017) who stated that the high discriminative value of MPV/PC ratio could predict severe ischemic stroke based on Rankin score ≥ 3 from a mild stroke. Also, Golcuk and colleagues (Golcuk et al. 2015) concluded that a combination of CURB-65 score and MPV can enhance the predictive accuracy of mortality in patients with acquired pneumonia.

## Conclusions

Once pneumonia develops in acute ischemic stroke patients, MPV/PC ratio could be considered as a significant laboratory marker.

## Abbreviations

COPD: Chronic obstructive pulmonary disease
CURB-65 score: Confusion, urea ≥ 7 mmol/L, respiratory rater ≥ 30 breaths/min, blood pressure ≤ 90 mmHg systolic or ≤ 60 mmHg diastolic, and age ≥ 65 years at pneumonia occurrence
CXR: Chest radiograph
GCS: Glasgow Coma Scale
MPV: Mean platelet volume
MPV/PC: Mean platelet volume to platelet count ratio
NIHSS: National Institutes of Health Stroke Scale
PSI score: Pneumonia severity index score
WBC: White blood cell
